# Development of a High-Efficiency Device for Thermal Neutron Detection Using a Sandwich of Two High-Purity ^10^B Enriched Layers

**DOI:** 10.3390/s23249831

**Published:** 2023-12-14

**Authors:** Chiara Provenzano, Marcella Marra, Anna Paola Caricato, Paolo Finocchiaro, Simone Amaducci, Fabio Longhitano, Maurizio Martino, Gaetano Elio Poma, Gianluca Quarta

**Affiliations:** 1 Department of Engineering of Innovation, University of Salento, 73100 Lecce, Italy; chiara.provenzano@unisalento.it; 2 National Institute of Nuclear Physics (INFN), 73100 Lecce, Italy; marcella.marra@unisalento.it (M.M.); annapaola.caricato@le.infn.it (A.P.C.); gianluca.quarta@unisalento.it (G.Q.); 3 Department of Mathematics and Physics “Ennio De Giorgi”, University of Salento, 73100 Lecce, Italy; maurizio.martino@unisalento.it; 4 INFN—Laboratori Nazionali del Sud, 95123 Catania, Italy; amaducci@lns.infn.it (S.A.); elio.poma@lns.infn.it (G.E.P.); 5 National Institute of Nuclear Physics (INFN)—Sezione di Catania, 95123 Catania, Italy; fabio.longhitano@ct.infn.it

**Keywords:** neutron detectors, solid state detectors, boron-10, helium-3 shortage, high-efficiency detector

## Abstract

The shortage of ${}^{3}$He, a crucial element widely used as a neutron converter in neutron detection applications, has sparked significant research efforts aimed at finding alternative materials, developing appropriate deposition methods, and exploring new detector architectures. This issue has required the exploration of novel approaches to address the challenges faced in neutron detection. Among the available conversion materials, ${}^{10}$B has emerged as one of the most promising choices due to its high neutron-capture cross-section and relatively high Q value. In our previous papers, we delved into the possibility of depositing neutron conversion layers based on ${}^{10}$B using Pulsed Laser Deposition (PLD). We investigated and evaluated the performance of these layers based on various factors, including deposition conditions, substrate properties, and film thickness. Moreover, we successfully developed and tested a device that employed a single conversion layer coupled with a silicon particle detector. In this current study, we present the development of a new device that showcases improved performance in terms of efficiency, sensitivity, and discrimination against γ background signals. The background signals can arise from the environment or be associated with the neutron field. To achieve these advancements, we considered a new detection geometry that incorporates the simultaneous use of two ${}^{10}$B conversion layers, each with a thickness of 1.5 μm, along with two solid-state silicon detectors. The primary objective of this design was to enhance the overall detection efficiency when compared to the single-layer geometry. By employing this novel setup, our results demonstrate a significant enhancement in the device’s performance when exposed to a neutron flux from an Am-Be neutron source, emitting a flux of approximately 2.2 × 10${}^{6}$ neutrons per second. Furthermore, we established a noteworthy agreement between the experimental data obtained and the simulation results.

## 1. Introduction

Neutrons, as uncharged particles, have unique characteristics that make them valuable for a wide range of applications in science, industry, and security. Among these, thermal neutrons (with kinetic energies around 0.025 eV) are particularly important in the framework of nuclear energy production, radioactive waste management, material analysis, and imaging. Detecting thermal neutrons accurately and efficiently requires specialized detectors. ${}^{10}$B-based thermal neutron detectors have emerged as powerful tools with versatile applications.

In addition to their role in nuclear power, neutron scattering research, and non-destructive testing, ${}^{10}$B-based thermal neutron detectors are useful in other domains—they are crucial in medical applications such as Boron Neutron Capture Therapy (BNCT), where they help deliver targeted cancer treatments by precisely monitoring the neutron flux. Furthermore, they contribute to security measures by monitoring potentially hazardous waste at customs checkpoints, ensuring public safety and environmental protection [1,2,3,4,5,6].

Overcoming challenges in detection efficiency, energy resolution, and background noise remain a focus of ongoing research. Advancements in detector materials, design optimization, and signal processing techniques will enhance the performance of ${}^{10}$B-based thermal neutron detectors, enabling further progress in science, industry, and security.

Neutron detection devices are developed through the combined influence of neutronics, the physical characteristics of neutron-sensitive materials, and the intended performance of the detector. Neutrons, as electrically neutral particles, cannot be directly detected by conventional detectors like scintillation, gas ionization, or solid-state detectors. To detect neutrons, they need to be converted into detectable charged particles. This conversion is achieved by using specific conversion elements that undergo reactions with neutrons, resulting in the emission of charged particles. When selecting a specific reaction, several considerations come into play [7]:

- Elements with a high reaction cross-section enable the construction of compact detectors.
- The target nuclide should either have a high natural isotopic abundance or be artificially enriched through cost-effective isotopic separation techniques.
- A reaction with a large Q-value must be chosen to ensure that the energy of the resulting secondary particle is sufficiently high for effective discrimination against signals induced by γ-rays.
- A reaction producing light particles is to be preferred, so that they can more easily cross possible dead layers of material before being detected.

Among the potential candidates as conversion elements, ${}^{6}$Li and ${}^{10}$B are excellent alternatives to ${}^{3}$He, which is commonly used but is facing decreasing availability [8,9,10]. Table 1 shows that ${}^{10}$B represents a good compromise between the energy of the reaction products, the Q-value, and the cross-section, although it produces capture products with lower energy as compared to ${}^{6}$Li. Therefore, our research efforts have focused on depositing thin films of ${}^{10}$B using Pulsed Laser Deposition (PLD).

In our previous study [11] we evaluated ${}^{10}$B conversion layers deposited at room temperature using PLD in terms of their performance when coupled with solid-state silicon detectors in a single-layer configuration. These micron-thick ${}^{10}$B films have demonstrated high uniformity, homogeneity, purity, and good mechanical properties. They adhere well to different substrates without delamination or cracking. To investigate their conversion properties, we deposited ${}^{10}$B films of varying thicknesses, ranging from 0.5 μm to 2.0 μm. We found that increasing the thickness of the ${}^{10}$B film improves the detection efficiency until it reaches a saturation point at around 2 μm. Indeed, for thicker films, the increased number of α and ${}^{7}$Li particles produced by neutron capture does not result in a larger detection efficiency, as those particles, produced deeper than 2 μm, reach the detector with energy below the detection threshold or are stopped inside the film. Monte Carlo simulations supported these findings, highlighting the necessity for smarter detection configurations to achieve enhanced intrinsic detection efficiency [12].

A significant challenge in neutron detection is the presence of background γ rays. These γ rays originate from neutron production processes or can be present in the particular environment. To address this challenge, techniques have been developed for the identification and discrimination of incident particles from the γ background. Various methods and algorithms, including the charge comparison method, zero crossing method, and pulse gradient analysis, have been studied [13,14,15,16]. These discrimination algorithms rely on the different signal shapes produced by the detector when hit by different incident radiations (γ or neutron) using a Pulse Shape Discrimination technique. Pulse Shape Discrimination is commonly employed with plastic scintillators [17,18,19,20]. Important contributions to background signal discrimination methods have also been made within the fields of Boron Neutron Capture Therapy (BNCT) and Neutron Capture Enhanced Particle Therapy (NCEPT) [21,22]. Additionally, machine learning techniques, such as clustering methods [23] and neural networks [24], have shown promise in discriminating neutron signals from overall signals. These methodologies typically rely on post-processing analysis and discrimination during the data processing phase rather than real-time during data acquisition. Our research goal is to develop an apparatus providing a mechanism for the removal of the γ radiation contribution in a single measurement.

In our previously tested geometry [11], the γ background contribution was subtracted by performing two measurements: the first using a silicon detector combined with the ${}^{10}$B converter layer, and the second using a bare silicon detector to record only the background radiation. By subtracting the two signals, we obtained the neutron spectrum.

In this paper, we introduce a new prototype that incorporates two ${}^{10}$B conversion layers, each 1.5 μm thick, and two identical double-sided silicon detectors in a sandwich configuration. This project aims to double the number of counts and at the same time more effectively take into account the contribution of γ rays. Furthermore, we have investigated the influence of different substrates, such as carbon fiber or aluminum, on neutron counts by configuring the same device using these two different substrates. It is important to emphasize that our employed background subtraction procedure is designed to eliminate possible spurious signals produced in the substrate.

## 2. Materials and Methods

### 2.1. PLD Deposition and ${}^{10}$B Films Characterization

Depositions by PLD of ${}^{10}$B conversion layers have been performed in a stainless-steel vacuum chamber, that has been evacuated down to 10${}^{-6}$ Pa thanks to a combined system of a rotative and a turbomolecular vacuum pumps [25].

**Figure 1:**
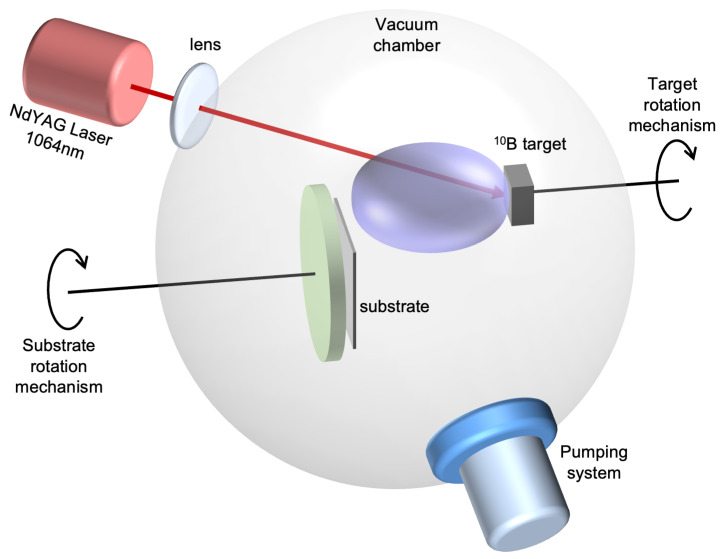
Pulsed Laser Deposition experimental setup.

The films have been deposited by ablating a commercial 95% isotopically enriched ${}^{10}$B target using the fundamental harmonic (1064 nm) of a Q-switched Continuum Powerlite-8010 Nd:YAG laser with a pulse width of 7 ns. The laser beam was focused on the ${}^{10}$B target at an incidence angle of 45${}^{\circ }$, producing a spot area of about 4 mm${}^{2}$. During the laser–target interaction, the ${}^{10}$B target underwent rotation at an angular speed of 1 Hz and simultaneous vertical movement. This was implemented to prevent the formation of craters due to multiple irradiations in the same area. The laser fluence has been set to 11 J/cm${}^{2}$, and laser repetition rate was fixed at 10 Hz. The experimental setup is reported in Figure 1.

The ablated material has been collected on a substrate placed in front of the target at a distance of 40 mm and with axis shifted with respect to the plume axis (off-axis configuration) held at room temperature.

The ${}^{10}$B layers have been deposited on two 50 × 50 mm${}^{2}$, 1 mm thick carbon fiber substrates and two similar aluminum substrates, growing a total of four identical ${}^{10}$B conversion layers 1.5 μm thick.

In previous studies the characterization of test samples carried out by Atomic Force Microscopy (AFM), Scanning Electrons Microscopy (SEM), X-ray Diffraction (XRD), and Rutherford Backscattering Spectrometry (RBS) analyses allowed us to carefully characterize the film properties [11].

### 2.2. Neutron Detection: Experimental Test Set-Up

Our detectors underwent testing by exposing them to a thermal neutron flux generated by an AmBe source emitting 2.2 × 10${}^{6}$ n/s, which was available at INFN-LNS in Catania, Italy. The source and its moderator were contained in a 95 × 75 × 85 cm${}^{3}$ iron box. Polyethylene and paraffin materials were utilized to surround the core, effectively thermalizing high-energy neutrons from several MeV down to 25 meV.

To ensure radiation protection, an outer shield composed of borated paraffin was employed, effectively intercepting most outgoing neutrons. An opening on the side of the box allowed for the placement of equipment to be exposed to the neutron flux. Remarkably, there was a huge gamma field inside the source box, due to the 34 GBq activity of ${}^{241}$Am which emits 59 keV gamma rays; due to the 2.2 MeV gammas from neutron capture in hydrogen; due to the 478 keV gammas emitted following neutron captures in the borated paraffin; and due to the 4.4 MeV gammas emitted along with the neutron after the (α,n) reaction on beryllium.

**Figure 2:**
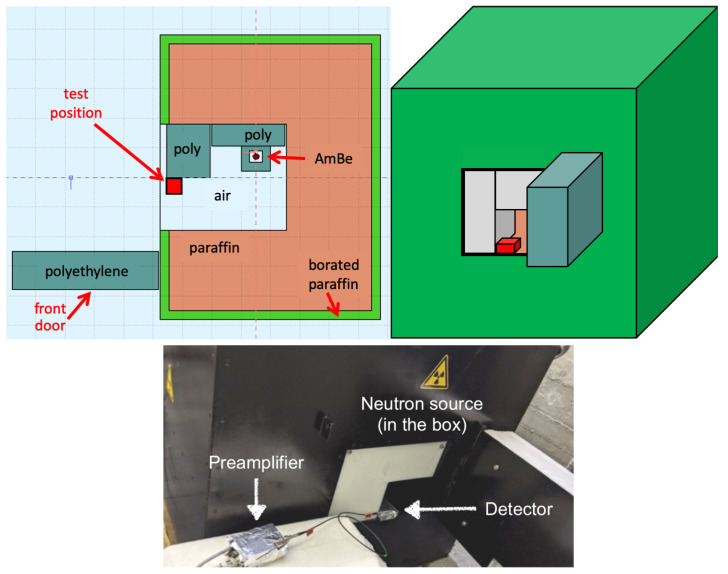
Top left: sketch of the mid-height section of the AmBe source box. Top right: 3D representation of the source box. Bottom: A picture of the AmBe neutron source with the detector in the test position.

Figure 2 illustrates a mid-height horizontal section, a 3D sketch, and a photograph of the AmBe neutron source with the detector in the testing position. By exploiting the AmBe source in conjunction with the designed setup, we were able to subject our detectors to a thermal neutron flux.

To maintain consistency in the measurements and replicate the same measurement conditions, all measurements were conducted in the same reference position. This approach was meant to minimize variations in the incident neutron flux, thereby ensuring a reasonable reproducibility and reliability of the experimental data. Unfortunately, we discovered that between the two sets of measurements described in Section 3.2, performed at different times, the internal position of the source and the arrangement of its surrounding polyethylene blocks had been slightly modified by other users. The net result was that the flux measurement, only performed after the second set of measurements, could not be reliably applied to the first one.

**Figure 3:**
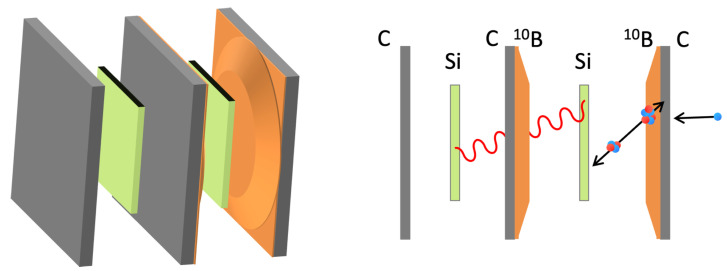
Sketch of the sandwich detector arrangement.

Two ${}^{10}$B conversion layers were coupled with two identical solid state silicon detectors [11]. The setup was reproduced identically, first testing the pair of conversion layers deposited on carbon fiber substrates and then later the layers deposited on aluminum. The solid state silicon detectors (MSX09-300 from Micron Semiconductor, 3 × 3 cm${}^{2}$ area and 300 μm thickness) have a 300 nm thick aluminum dead layer on each face acting as electric contact. The tested geometry is sketched in Figure 3.

The inner silicon detector faces both ${}^{10}$B converter layers, whereas the outer silicon detector only faces substrates. In this configuration, only the first silicon detector receives alphas and ${}^{7}$Li particles generated in the ${}^{10}$B converters, while both silicon detectors are reached by the background γ radiation, whose contribution can then be measured and subtracted.

Each detector was biased at 30 V, the output signal was handled by an ORTEC 142B preamplifier followed by a an ORTEC 672 amplifier. The analog-to-digital conversion and the spectra have been obtained by using a Dual Digital MCA CAEN Mod. DT5781A digitizer.

**Figure 4:**
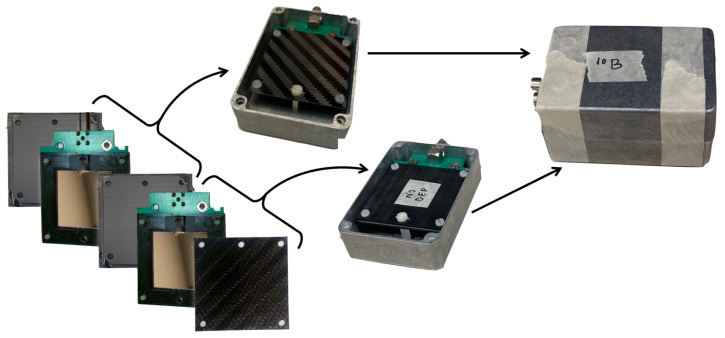
Construction phases of the detector prototype: A silicon detector was assembled between two carbon fiber layers with ${}^{10}$B conversion films on the inner faces. Another identical silicon detector was coupled with a bare substrate. Both packages were boxed and assembled according to the scheme shown in Figure 3. The same setup was also assembled using aluminum substrates.

Figure 4 shows the final sandwich device and the steps followed to assemble it. The same procedure was followed to assemble two devices, by using carbon fiber or aluminum as ${}^{10}$B substrates.

## 3. Results and Discussion

### 3.1. ${}^{10}$B Layers Properties

${}^{10}$B films were successfully deposited on carbon fiber and aluminum substrates, resulting in uniform layers covering a circular area of approximately 30 mm. Further details about the film properties can be found in reference [11]. The deposition process was carried out at room temperature, and the films exhibited excellent adhesion to the substrates, without any signs of delamination, cracking, or peeling-off events.

**Figure 5:**
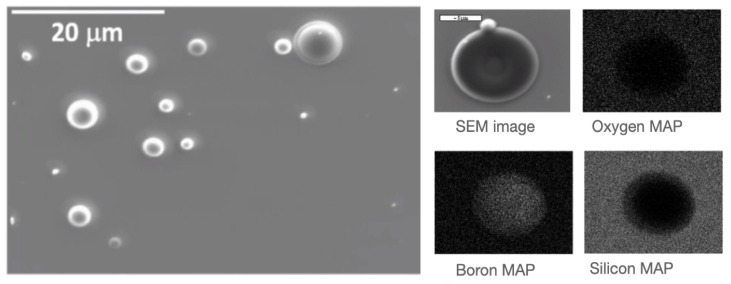
(**Left**) Secondary electrons SEM image of a ${}^{10}$B film (200 nm) deposited on Si/SiO${}_{2}$ substrate by PLD. (**Right**) BSE image of a droplet with the elemental analysis map on the droplet. The oxygen signal is only due to the substrate.

Scanning Electron Microscopy (SEM) and Atomic Force Microscopy (AFM) images revealed a continuous layer, with the exception of micro and nano-aggregates. These aggregates were formed due to the deposition of molten droplets ejected from the target during the ablation process, known as explosive boiling. Despite the presence of these aggregates, the films exhibited good overall integrity and coverage (Figure 5).

To assess the film purity and density, Rutherford Backscattering Spectrometry (RBS) and Energy-Dispersive X-ray Spectroscopy (EDS) analyses were conducted. The results indicated the absence of oxygen contamination within the ${}^{10}$B layer deposited by Pulsed Laser Deposition (PLD), while the density values were found to be close to the nominal values. X-ray Diffraction (XRD) spectra revealed that the films were amorphous, with a certain degree of order attributed to the presence of icosahedral units.

Overall, the characterization results demonstrated that the deposited ${}^{10}$B films were highly stable and possessed favorable properties for their application in neutron detector devices.

### 3.2. Measurements of Neutron Counts

In our previous work, a single ${}^{10}$B conversion layer was coupled with a silicon detector. With this configuration, in order to take into account the γ background, a double measurement was performed: (i) “face to face” measurement—the ${}^{10}$B conversion layer was placed at 200 μm in front of the silicon detector; (ii) “back to face” measurement—the conversion layer was turned upside down with respect to the detector, placing the substrate in front of it, in order to register only the background γ radiation. In this way, the substrate thickness prevented ${}^{7}$Li and α particles from reaching the silicon [11]. The final deposited energy spectrum with the only contribution from neutrons was then obtained by subtracting the “back to face” spectrum from the “face to face” one.

The influence of the substrate on neutron detection was assessed using a single layer configuration. To this end, conversion layers were deposited on either carbon fiber or aluminum, and each substrate was coupled individually to the same silicon detector.

These single-layer devices were exposed to the neutron source. The deposited energy spectra were collected in a range spanning from ∼0.2 to 1.8 MeV. The obtained spectra exhibit similar characteristics, indicating the consistent performance of the two devices despite being assembled with different substrates. Specifically, the neutron counts demonstrate comparable values, considering both the uncertainties associated with the measurement and the 10% uncertainty in the thickness of ${}^{10}$B films. In this single-layer configuration, the neutron count, obtained by integrating the spectrum from 0.2 MeV to 1.8 MeV, was 7.73 ± 0.19 n/s when utilizing the film with a C-fiber substrate and 7.88 ± 0.18 n/s for the film deposited on aluminum.

In a subsequent run the new sandwich devices were tested, employing the same geometric configuration with both carbon fiber and aluminum substrates, and tested at the same position inside the neutron source box.

**Figure 6:**
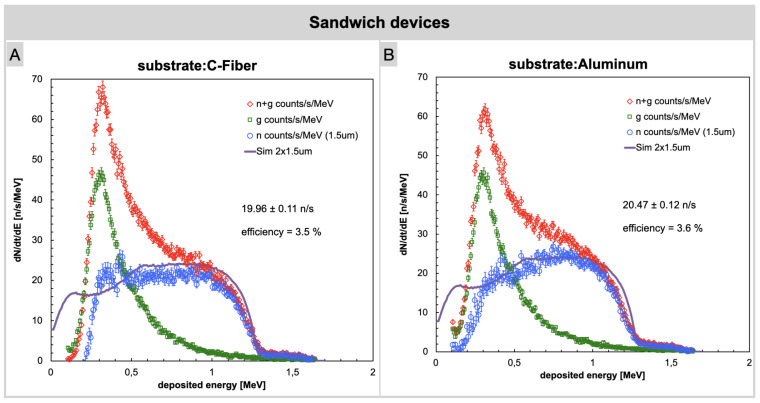
Neutron counts acquired using sandwich devices. (**A**) Carbon fiber substrate. (**B**) Aluminum substrate.

Figure 6 illustrates the spectra obtained using sandwich devices for carbon fiber (left) and aluminum (right) substrates. Interestingly, a slightly different spectral shape was observed between the two configurations, which might be related to slight irregularities in the converter layers. However, the overall detection efficiency was not affected and therefore one can state that the substrate material does not influence the neutron detection efficiency of the device.

In this experimental configuration a significant increase in the neutron count rate was observed as compared to the previous measurements, as expected due to the double converter layers. Alpha particles and gamma background were counted on one detector while the γ background only, to be used for subtraction, was acquired simultaneously on the second detector. This approach guarantees a more accurate background subtraction. We remark that given the thickness of the involved layers of converter, air, and aluminum the ${}^{7}$Li ions could barely reach the silicon with enough kinetic energy to produce a useful signal; therefore, their contribution was neglected.

The net neutron count for the sandwich devices, observed within the 0.2 MeV to 1.8 MeV energy range, increases to 19.96 ± 0.11 n/s and 20.47 ± 0.12 n/s for the sandwich with ${}^{10}$B layers deposited on C-fiber and aluminum substrates, respectively. The significant enhancement in the measured neutron count rate is obviously attributed to the doubling of the ${}^{10}$B converter layers, which allows for a higher number of conversions and, consequently, an increased number of α particles impacting the silicon detector. It is important to note that the neutron flux is slightly attenuated after crossing one converter layer and then reaching the other one. This was taken into account in the simulation as well. To validate the experimental findings, simulations were performed using the Geant4 software. The simulation results confirm the observed trends in the data, providing additional support for the experimental measurements and strengthening the overall credibility of the study results. The simulated plot in Figure 6 is derived from the simulation of alpha particles. It is worth noting that the agreement between the experimental plot and the simulation is not perfect. This deviation arises from various sources of uncertainty. In addition to considering statistical uncertainty, it is necessary to account for the variability in the output flux from the source due to differences in the geometric positioning of the detector relative to the source. Furthermore, uncertainties related to the thickness of the film and its non-uniformity should also be taken into account. However, it is essential to emphasize that the sum of net neutron counts obtained by integrating the experimental curve aligns quite well with the sum of simulated counts in both cases, within the specified energy range of 0.2 MeV to 1.8 MeV. The discrepancy observed is solely in the spectrum shape near the lower energy values.

At the end of the second run we performed a flux measurement by placing a reference detector, featuring an identical silicon diode coupled to a single 1.6 µm ${}^{6}$LiF converter layer on carbon fiber that had been previously calibrated at a certified thermal neutron flux at the same measurement position [26]. The measured flux, from the counted 720 ± 9 neutrons per second on the 9 cm${}^{2}$ silicon area, was 80 ± 1 n/s/cm${}^{2}$. The resulting effective flux, by scaling the incoming neutrons to about 600 per second due to self absorption in either one of the two converters of the sandwich, was about 9 neutrons/s/cm${}^{2}$. This final value contains some intrinsic uncertainties, not easily quantifiable, which though affecting the final value of the detection efficiency still allow for a reasonable estimate. Table 2 lists the reported neutron counts obtained by integrating the measured deposited energy spectra from 0.2 to 1.8 MeV for the four tested devices. This table provides a summary overview of the performance of our detectors, allowing for a concise comparison of their respective capabilities. To assess the detection efficiency, we calculated the ratio of the integral of the blue curve (representing the net neutron count) to the neutron flux reaching the detector. This approach allows for a quantitative evaluation of the device capability to capture and measure neutron events.

## 4. Conclusions

Building upon prior research expertise and experimental foundations, we have achieved the successful development of a technological solution that significantly enhances detection efficiency and sensitivity while effectively mitigating gamma (γ) background signals. The core innovation lies in the implementation of a “sandwich-like” structure, comprising two high-purity ${}^{10}$B enriched layers deposited through Pulsed Laser Deposition (PLD) and integrated with two identical solid-state silicon detectors. Notably, the neutron counting efficiency doubled in comparison to previous devices that relied on a single layer. This substantial increase in the neutron count rate, as anticipated due to the inclusion of double converter layers, has to be attributed to a greater number of conversions and alpha particles impacting the silicon detector. This enhancement is paramount for elevating the precision of neutron detection. Although this upgrade incurred heightened costs associated with the use of two ${}^{10}$B conversion films and detectors, the superior system performance justifies this supplementary investment. Simulation results using the Geant4 software supported the experimental findings, aligning with the observed trends, while some deviation was noted, mainly in the spectrum shape near lower energy values; this can be attributed to various sources of uncertainty, such as source flux variability and film thickness.

Furthermore, we investigated the influence of different substrates (specifically aluminum and carbon fiber) on neutron counts. The results indicated that the choice of substrate resulted in a slightly different spectral shape but this variation did not impact overall detection efficiency and the signal intensity remained consistent. In summary, this experiment introduces an innovative approach to thermal neutron detection, offering amplified detection efficiency, heightened sensitivity, and improved discrimination against γ background signals. The employment of the sandwich-like structure marks a significant stride in the field, furnishing a practical solution for researchers and industries reliant on precise neutron detection. These findings bear substantial potential for the advancement of scientific and practical applications dependent on accurate neutron measurements.

Various potential alternatives are currently being explored in the scientific literature, including the utilization of alternative conversion materials and technologies for particle detection [27]. When evaluating the performance of the proposed ${}^{10}$B-based detectors in comparison to existing solutions, we anticipate a cost reduction through economies of scale. It is noteworthy that the high efficiencies reported in the literature mostly pertain to 3D detectors, which involve considerably higher manufacturing costs. Looking ahead, we also envision enhancing detection performance by incorporating Pulse Shape Discrimination approaches to mitigate gamma background.

## Figures and Tables

**Table 1:** Neutron conversion reactions.

Species	Reaction	Q (MeV)	E Products (MeV)	σ (Barn)
${}^{3}He$	${}^{3}He$+*n*→${}^{3}H$+*p*	0.764	$E_{p}=0.57$, $E_{H}=0.19$	5320
${}^{10}B$	${}^{10}B$+*n*→${}^{7}Li$+α	2.792 (6%)	$E_{\alpha }=1.78$, $E_{Li}=1.01$	230
${}^{10}B$	${}^{10}B$+*n*→${}^{7}Li^{*}$+α+γ	2.310 (94%)	$E_{\alpha }=1.47$, $E_{Li}=0.84$	3607
${}^{6}Li$	${}^{6}Li$+*n*→${}^{3}H$+α	4.780(6%)	$E_{\alpha }=2.05$, $E_{H}=2.73$	942

**Table 2:** Sum of the net neutron count from 0.2 to 1.8 MeV per second and detection efficiency of the devices.

Device	Substrate	Sum [counts/s]	Detection Efficiency
Single layer of ${}^{10}$B	C-fiber	7.73 ± 0.19	no reference flux
Single layer of ${}^{10}$B	Aluminum	7.88 ± 0.18	no reference flux
Sandwich of two ${}^{10}$B layers	C-fiber	19.96 ± 0.11	3.5%
Sandwich of two ${}^{10}$B layers	Aluminum	20.47 ± 0.12	3.6%

## Data Availability

The data presented in this study are available on request.
